# Growth mixture modeling as an exploratory analysis tool in longitudinal quantitative trait loci analysis

**DOI:** 10.1186/1753-6561-3-s7-s112

**Published:** 2009-12-15

**Authors:** Su-Wei Chang, Seung Hoan Choi, Ke Li, Rose Saint Fleur, Chengrui Huang, Tong Shen, Kwangmi Ahn, Derek Gordon, Wonkuk Kim, Rongling Wu, Nancy R Mendell, Stephen J Finch

**Affiliations:** 1 Department of Applied Mathematics and Statistics, Stony Brook University, 100 Nicolls Road, Stony Brook, New York 11794, USA; 2 Department of Public Health Sciences, Pennsylvania State College of Medicine, Pennsylvania 17033, USA; 3 Department of Genetics, Rutgers University, 145 Bevier Road, Piscataway, New Jersey 08854, USA; 4 Department of Mathematics and Statistics, University of South Florida, 4202 East Fowler Avenue, Tampa, Florida 33620, USA

## Abstract

We examined the properties of growth mixture modeling in finding longitudinal quantitative trait loci in a genome-wide association study. Two software packages are commonly used in these analyses: Mplus and the SAS TRAJ procedure. We analyzed the 200 replicates of the simulated data with these programs using three tests: the likelihood-ratio test statistic, a direct test of genetic model coefficients, and the chi-square test classifying subjects based on the trajectory model's posterior Bayesian probability. The Mplus program was not effective in this application due to its computational demands. The distributions of these tests applied to genes not related to the trait were sensitive to departures from Hardy-Weinberg equilibrium. The likelihood-ratio test statistic was not usable in this application because its distribution was far from the expected asymptotic distributions when applied to markers with no genetic relation to the quantitative trait. The other two tests were satisfactory. Power was still substantial when we used markers near the gene rather than the gene itself. That is, growth mixture modeling may be useful in genome-wide association studies. For markers near the actual gene, there was somewhat greater power for the direct test of the coefficients and lesser power for the posterior Bayesian probability chi-square test.

## Background

Growth mixture modeling (GMM) is an important tool for analyzing longitudinal data [1-3]. GMM hypothesizes that there is a fixed but unknown number of trajectory pattern components observed in a population. GMM applies mixture analysis methods to estimate the number of trajectory components and the probability that a trait variable (such as a genotype) affects the probability of trajectory component membership. The procedure allows for controlling for time-varying covariates (TVCs) as well. Two popular software packages for GMM are the SAS TRAJ procedure [4-6] and the Mplus program [7-9]. We analyze simulated data on the coronary artery calcification (CAC) measurements taken at the three visits [10]. We apply GMM software to assess whether genotypes appear to be associated with trajectory component membership, and hence identify longitudinal quantitative trait loci (QTL). We compare the empirical distribution of three measures of association for genes not in the genetic model for CAC to the usual chi-squared distributions. We compare the power of GMM analyses that explicitly incorporate genotype measurements of the genes in the genetic model for CAC into the mixture modeling to GMM analyses that assess genetic association with post hoc tests. Finally, we also report the reduction in power using genes close to the true gene rather than the gene itself.

## Methods

### Analysis software

We use the SAS TRAJ procedure [6] and the Mplus program [11] to perform GMM and to identify longitudinal QTLs. Each SAS TRAJ analysis reports the maximized log-likelihood, the maximum-likelihood estimates (MLEs) of the trajectory group parameters, the *t*-statistics of the trajectory group parameters, the estimated frequency of each trajectory group, the Bayesian posterior probability (BPP) that each subject is a member of each trajectory component and the Bayesian information criterion (BIC) statistic, which is used to assess the number of trajectory components. Mplus also reports these statistics.

### Genes used in the analysis

For each gene considered, we create the two indicator variables, whether the subject's genotype is the more common homozygote and whether the subject's genotype is the less common homozygote. We study 17 single-nucleotide polymorphisms (SNPs): *τ*_1_, ..., *τ*_5_, *ϕ*_1_, *ϕ*_2 _and 10 SNPs *v*_1_, *v*_2_, ..., *v*_10 _on human chromosome (HC) 22 that were not in the genetic mechanism determining the simulated CAC and myocardial infarction (MI) events. The genes *τ*_1_, ..., *τ*_5 _determined the CAC level, and the genes *ϕ*_1_, *ϕ*_2 _determined MI but not the CAC level [10]. The results for *v*_1_, *v*_2_, ..., *v*_10 _are the basis of the empirical null distribution of our test statistics. The distribution for *ϕ*_1_, *ϕ*_2 _should be similar to the empirical null distribution. We also report PROC TRAJ results for four SNPs near *τ*_5 _and *τ*_2 _that had a minor allele frequency (MAF) greater than 0.1 and were in Hardy-Weinberg equilibrium (HWE) to demonstrate the applicability of this procedure for genome-wide association studies (GWAS).

### CAC analyses

We consider two sets of analyses applied to the 200 replicates. For each of the 17 SNPs, the first uses the longitudinal CAC measures with the two genetic indicator variables used as traits but without any TVCs. The second is the longitudinal CAC with the TVCs cholesterol (CHOL) and high-density lipoprotein (HDL) level and with the two genetic indicator variables as traits. We use a quadratic trend function and set the number of components to two and three. The dataset consists of subjects with genotypes and simulated phenotypes (*n *= 6,476). We treat the subjects as independent observations.

### Measures of association with a gene

In an analysis that identifies *c *trajectory groups, there are 2(*c*-1) indicator variables associated with gene *i*, *i *∈ {*τ*_1_, ..., *τ*_5_, *ϕ*_1, *ϕ*2_, *v*_1_, ..., *v*_10_}. For example, for the *τ*_5 _gene (which has homozygous genotypes *AA *and *GG*), there are estimated coefficients for the two homozygous indicators in Groups 2 through *c*. Group 1 is a reference group with coefficients of trait variables set to 1 identically in the SAS TRAJ procedure. With *τ*_5_, we calculate  and approximate its null distribution with the empirical distribution for *v*1, *v*2, ..., *v*_10_. We call this the "direct coefficient test" (DCT) and use level of significance 0.05 with the empirical critical value from the distribution for *v*_1_, *v*_2_, ...., *v*_10_.

Our second procedure is the BPP chi-squared test on the three genotype rows by *c *trajectory group column contingency table. We classify each subject into the trajectory group that has the largest BPP. A significant value of the chi-square test for independence (*p *< 0.05 based on the empirical distribution of the chi-square test for *v*_1_, *v*_2_, ...., *v*_10_) indicates association with the gene.

Our third procedure is the likelihood-ratio test statistic (LRTS). We take the difference of the likelihood function with the two genetic indicator variables and the likelihood function without the two genetic indicator variables. We perform this test without TVC and with TVC. A significant value of the LRTS (*p *< 0.05 based on the distribution of the chi-square test for *v*_1_, *v*_2_, ...., *v*_10_) indicates association with the gene.

## Results

We ran the Mplus software on Replicates 1 through 11 with two and three trajectory groups specified, with subject's age as individually varying times of observations for the outcome CAC. The software either failed to converge or failed to identify the solution due to excessive numbers of local maxima. We used at least 500 sets of starting values in the initial stage and 100 optimizations in the second stage. Computation times were between 67 and 75 hours for each replicate to fit the two-group models without any time-invariant or time-varying covariates. The Mplus software was not considered any further.

The distribution of the results from the three procedures using the SAS TRAJ procedure had greater means and standard deviations for the five SNPs from *v*_1_, *v*_2_, ...., *v*_10 _that were not in HWE than for the five in HWE as shown in Table 1. We used the 95^th ^percentile for the five markers in HWE as the critical value for our tests. Use of TVC appeared to increase the mean and standard deviation of the distribution.

**Table 1:** Summary test statistics for the HC22 SNPs, 200 replicates

			Mean (SD)		
				
Test	Group	TVC	In HWE	Not in HWE	In HWE, 95^th ^percentile
LRTS	2	no	699.07 (929.04)	20112.96 (9897.54)	2547.31
LRTS	2	yes	14284.71 (921.18)	32726.20 (9359.47)	16184.79
LRTS	3	no	698.58 (925.06)	20044.89 (9853.60)	2535.54
LRTS	3	yes	14722.74 (928.14)	32905.40 (9241.30)	16618.77
					
DCT	2	no	1.22 (1.24)	2.87 (3.06)	3.81
DCT	2	yes	1.62 (1.52)	3.40 (3.74)	4.72
DCT	3	no	3.48 (3.17)	4.24 (3.31)	9.27
DCT	3	yes	4.04 (5.82)	3.97 (3.17)	10.48
					
BPP	2	no	2.20 (2.27)	4.82 (5.52)	6.64
BPP	2	yes	2.95 (2.80)	8.07 (15.13)	8.46
BPP	3	no	6.32 (5.14)	8.88 (7.12)	16.74
BPP	3	yes	9.26 (10.10)	14.91 (19.20)	22.42

For *ϕ*_1_, *ϕ*_2 _and *τ*_1_, ..., *τ*_5_, we studied either two- or three-trajectory components, with and without TVCs. Table 2 contains rejection rates by gene for the three tests using the two- and three-trajectory group models. For *ϕ*_1 _and *ϕ*_2_, which are genes associated with MI but not CAC, the DCT and BPP rejection rates are roughly consistent with 5% level of significance. The LRTS rejection rates are 0, suggesting that the test is not well defined for this application, and we did not consider the LRTS further. For *τ*_5_, the rejection rate was 100% for both DCT and BPP using the two-trajectory group model. The corresponding rejection rate for *τ*_2 _is 97.5% for DCT and BPP. For *τ*_1_, *τ*_3_, and *τ*_4_, the rejection rates for DCT and BPP are not substantially above 5%, the level of significance.

**Table 2:** Rejection rates of tests by gene, 200 replicates

	2 Groups		3 Groups	
	
Gene, test	No TVC	TVC	No TVC	TVC
*ϕ*_1_				
LRTS	0	0	0	0
DCT	7.5	10.5	2.5	9.0
BPP	18.0	10.0	4.5	10.5
*ϕ*_2_				
LRTS	0	0	0	0
DCT	4.0	1.0	3.0	2.0
BPP	3.0	2.5	1.0	1.5
*τ*_5_				
LRTS	0	0	0	0
DCT	100	100	90.0	85.0
BPP	100	100	90.0	85.0
*τ *_2_				
LRTS	0	0	0	0
DCT	97.5	100	65.5	85.5
BPP	97.5	100	80.5	85.5
*τ *_1_				
LRTS	0	0	0	0
DCT	12.5	3.5	1.5	30.0
BPP	9.5	1.0	1.5	16.5
*τ *_3_				
LRTS	0	0	0	0
DCT	2.5	1.0	3.5	3.0
BPP	3.0	2.0	3.5	3.0
*τ *_4_				
LRTS	0	0	0	0
DCT	1.5	6.5	1.0	3.5
BPP	2.0	3.0	0.5	0

**Figure 1:**
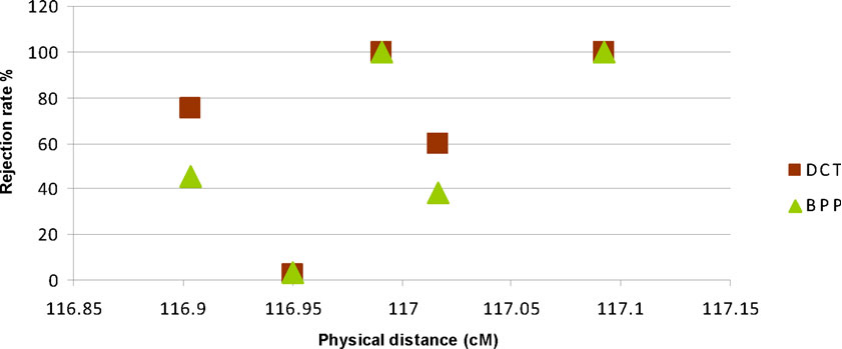
**Rejection rate of DCT and BPP for SNPs near *τ*_5 _by position with *τ*_5_**. The empirically obtained critical values were used (see Table 1, column 4).

Figure 1 shows the rejection rate of DCT and BPP for four SNPs near *τ*_5 _(116.99 cM). The rejection rate for one of the nearby SNPs was 100% for both DCT and BPP, and the rejection rate was greater than 50% for DCT for two nearby SNPs. The rejection rate for the remaining SNP was very low. The rejection rate for BPP was somewhat less than the rejection rate for DCT. Similar results held for *τ*_2 _(data not shown).

## Discussion and conclusion

The Mplus software was not effective in analyzing this data due to computational instability and lengthy computing time. Computational instability also affected the SAS TRAJ procedure. For example, about 17% of the replicates did not have a solution when three groups were specified with TVCs. One effect of this instability was that using TVCs did not increase the overall power as expected. The LRTS was not usable, possibly due to the dependence of subjects within pedigree and the non-normality of the distribution of CAC, especially for the first visit. The empirical distribution of the DCT and BPP for genes not associated with CAC values appeared to depend on whether the gene was apparently in HWE. Because violation of HWE is often used as a test for large genotyping error rates [12], a question yet to be resolved is the robustness of these procedures to genotyping error. A second approach to calculating the *p*-value of the DCT or BPP is to use a permutation method.

Specifically, one can generate a large number of random permutations of the *n *vectors (here *n *= 6,476 participants) of CAC values. The fraction of permutations that yield a value of the statistic larger than the one observed is the permutation *p*-value.

The analyses based on the SAS TRAJ procedure have power to detect genes associated with longitudinal for CAC QTLs. For CAC, the SAS TRAJ analysis of unadjusted CAC values with two trajectory groups and no TVCs had excellent power (100% rejection rate) to detect the *τ*_5 _association and good power (97.5% rejection rate) to detect the *τ*_2 _association using either DCT or BPP. The associations with *τ*_1_, *τ*_3_, and *τ*_4 _were not detected with this approach. The rejection rates at a marker near *τ*_5 _could be as large as the *τ*_5 _rejection rate. The rejection rate for DCT was high for most markers near *τ*_5_. The BPP had somewhat lower rejection rates than the DCT. Similar results held for *τ*_2_.

We conclude that the SAS TRAJ procedure is useful in GWAS to identify longitudinal QTLs. In an actual genetic analysis, one should follow Maclean et al. and consider multiple transformations of the data to reduce the chances that skewness of the data would result in an apparent genetic association [13]. Procedures to find the most effective transformation should be developed to enhance the applicability of GMM analysis. The chi-square test using the Bayesian posterior probability classification of subjects seems to be slightly less powerful than the direct test of the coefficients.

## List of abbreviations used

BIC: Bayesian information criterion; BPP: Bayesian posterior probability; CAC: Coronary artery calcification; DCT: Direct coefficient test; GMM: Growth mixture modeling; GWAS: Genome-wide association studies; HC: Human chromosome; HWE: Hardy-Weinberg equilibrium; LRTS: Likelihood-ratio test statistic; MAF: Minor allele frequency; MI: Myocardial infarction; MLE: Maximum likelihood estimate; QTL: Quantitative trait loci; SNP: Single-nucleotide polymorphism; TVC: Time-varying covariate.

## Competing interests

The authors declare that they have no competing interests.

## Authors' contributions

S-WC developed the methodology, carried out the statistical and genetic analyses, organized the group, and drafted the manuscript. SHC, KL, RSF, CH, and TS participated in the data mining and statistical analyses. KL was also in charge of the data distribution and management. KA, DG, WK, RW, NRM, and SJF conceived of statistical theories and possible applications. NRM and SJF also participated in the study design, group coordination, and manuscript drafting. All authors read and approved the final manuscript.
